# Neuronal Populations Involved in Motor Function Show Prominent Expression of Sbno1 During Postnatal Brain Development

**DOI:** 10.3390/jdb13010003

**Published:** 2025-01-21

**Authors:** Sunjidmaa Zolzaya, Dai Ihara, Munkhsoyol Erkhembaatar, Shinsuke Ochiai, Ayaka Isa, Mariko Nishibe, Jean-Pierre Bellier, Takahiro Shimizu, Satoshi Kikkawa, Ryo Nitta, Yu Katsuyama

**Affiliations:** 1 Department of Anatomy, Division of Neuroanatomy, Shiga University of Medical Science, Otsu 520-2192, Japan; sunjidma@belle.shiga-med.ac.jp (S.Z.); daiihara@belle.shiga-med.ac.jp (D.I.); munkhsoyol@mnums.edu.mn (M.E.);; 2 Molecular Neuroscience Research Center, Shiga University of Medical Science, Otsu 520-2192, Japan; 3 Department of Neurology, Brigham and Women’s Hospital/Harvard Medical School, Boston, MA 02115, USA; 4 Department of Physiology and Cell Biology, Division of Structural Medicine and Anatomy, Kobe University Graduate School of Medicine, Kobe 650-0017, Japan

**Keywords:** postnatal brain development, brainstem motor nuclei, Sbno1

## Abstract

Human genome studies have suggested that strawberry notch homologue 1 (*SBNO1*) is crucial for normal brain development, with mutations potentially contributing to neurodevelopmental disorders. In a previous study, we observed significant developmental abnormalities in the neocortex of *Sbno1* as early as one week after birth. In the present study, we conducted an extensive analysis of Sbno1 postnatal expression in the brain of C57BL/6 mice using a newly developed in-house polyclonal antibody against Sbno1. We found that Sbno1 is expressed in all neurons, with certain neuronal populations exhibiting distinct dynamic changes (both temporal and spatial) in expression level. These findings suggest that the neuronal expression of Sbno1 is developmentally regulated after birth. They also indicate that while Sbno1 may play a general role across all neurons, it may also serve more specialized functions in certain neuronal types and/or for certain cellular activities related to particular neuronal pathways.

## 1. Introduction

The genetic studies of patients and their families, along with the occurrence of similar symptoms in twins, suggest a genetic risk for neurodevelopmental disorders, such as autism and schizophrenia [1,2]. Girard et al. examined de novo mutations in eight schizophrenic probands, finding mutations in fifteen genes, including *SBNO1* [3]. Other studies have suggested a link between schizophrenia and *SBNO1* [4,5]. Head circumference measurement, together with conventional anthropometry, is essential for assessing neurodevelopment in infants [6]. A genome-wide association study (GWAS) involving 10,768 participants was conducted to identify gene loci associated with deviations in head circumference during infancy, resulting in the identification of two loci (12q15 and 12q24) that significantly accumulated single nucleotide polymorphisms (SNP). Interestingly, *SBNO1* is located in 12q24 [7]. *SBNO1* has also been reported to be among genes associated with intellectual abnormalities [8]. Studies in zebrafish have shown that *SBNO1* deficiency leads to brain abnormalities [9]. These findings suggested the essential role of *SBNO1* in normal brain development, indicating that mutations in *SBNO1* may serve as a pathogenetic factor in neurodevelopmental disorders.

*Sbno1* is one of the vertebrate homologues of the Drosophila gene *strawberry notch* (*sbno*). The phenotype of *sbno* mutants in flies exhibits notched wings and disorganized eyes, referred to as the *strawberry* phenotype [10]. These and other abnormalities in *sbno* mutants overlap with those observed in Notch signaling-related mutants, suggesting that the sbno protein is a component of the Notch signaling pathway. Nevertheless, the *sbno* mutant of Drosophila did not disrupt the lateral inhibition event in neuronal differentiation, leading to the conclusion that *sbno* may play a role in the specification between lateral inhibition and inductive Notch signaling pathways [11]. Subsequently, Tsuda et al. [12] demonstrated that sbno biochemically binds to the Su(H) transcription factor, which regulates the expression of the *delta* gene, encoding for a ligand of Notch. Su(H) is the homolog of vertebrate Rbpj, and we have found that sbno1 binds to Rbpj through yeast two-hybrid screening [13].

We previously examined the expression of the Sbno1 protein in the neocortex during mouse embryonic development, whereas developmental abnormalities in Sbno1 knockout (KO) mice became apparent one week after birth [14]. In this report, we investigated the postnatal expression of the Sbno1 protein in the C57BL/6 mouse brain. For this purpose, we developed a new polyclonal antibody against the Sbno1 protein. Notably, all neurons express Sbno1; however, its expression was prominent in brain structures associated with motor function, such as layer V of the cerebral cortex, motor nuclei in the brainstem, and sporadically distributed neurons in the reticular formation. These observations suggest that Sbno1 functions in all neurons, while playing a unique role in specific neuronal populations.

## 2. Materials and Methods

### 2.1. Antigen Preparation

cDNA sequence coding for the amino acid sequence of the *N*-terminus of human SBNO1 (amino acid 13-130) was incorporated into the pET-21b (+) DNA vector (Merck). The vector was subsequently incorporated into competent *E. coli* BL21(DE3) cells. Transformed competent cells were cultured in 5 L of lysogeny broth (LB) medium supplemented with Ampicillin at 37 °C under shaking conditions until the optical density at 600 nm (OD_600_) reached 0.6. The cells were then treated with isopropyl-β-D-1-thiogalactopyranoside at a final concentration of 0.1 mM and incubated at 37 °C for 24 h. Following centrifugation, the cell pellets were resuspended in 100 mL of washing buffer (50 mM Na-phosphate buffer, pH 8.0, containing 0.5 M guanidine HCl). After centrifugation and removal of the supernatant, the pellets were dissolved by sonication in extraction buffer at the concentration of 0.1 g/mL (50 mM Na-phosphate buffer, pH 8.0, containing 2.2 M guanidine hydrochloride). Imidazole was supplemented to the post-centrifugation supernatant at a final concentration of 20 mM and subsequently applied to a pre-equilibrated column filled with HIS-Select Nickel resin (Sigma-Aldrich, St. Louis, MI, USA). Wash buffer was applied to the column until the absorbance at OD_280_ of the buffer wash through the column fell below 0.1. Then, protein was eluted using elution buffer (extraction buffer complemented with 200 mM imidazole). The sample was dialyzed in 100× volume of refolding buffer (20 mM Na-phosphate buffer (pH 8.0), 0.3 M NaCl, 2 mM DTT) (Figure S1 lane 1). HRV 3C protease was added to the sample to remove the His tag, which was sequentially passed through Ni resin and glutathione sepharose resin, and the fraction passing through was collected as the final antigen. The obtained recombinant human Sbno1 fragment was run on acrylamide gel electrophoresis and stained by Coomassie Brilliant Blue for quality control (Figure S1 lane 2).

### 2.2. Generation of Polyclonal Antibody

An 8-week-old rabbit (Slc: NZW) was immunized via subcutaneous injection with the recombinant human Sbno1 fragment conjugated to Keyhole Limpet Hemocyanin (KLH) (Thermo Fisher Scientific, Waltham, MA, USA). In total, 50 mg of KLH conjugated to the antigen was dissolved in 150 μL of Complete Freund’s Adjuvant (Sigma-Aldrich # F5881). The antigen was injected into multiple sites on the rabbit’s back skin with a 2-week interval between each boost injection. Following 12 injections, the rabbit was deeply anesthetized using sodium pentobarbital, and the whole blood was collected from the rabbit’s heart into a serum-separating vacutainer (Nipro Inc., Osaka, Japan). The rabbit was euthanized by intracardiac injection of a lethal dose of sodium pentobarbital. After centrifugation of the blood containing vacutainer, the isolated serum was diluted with an equal volume of glycerol and stored in the freezer until use.

### 2.3. Animals

C57BL/6 mice were maintained on a 12:12 h light and dark cycle in a temperature-controlled breeding room (21 °C) and were naturally mated to obtain pups. The day the pups were born was designated as postnatal day 0 (P0). The pups were anesthetized with isoflurane inhalation solution (219KAT, Pfizer, New York, NY, USA) using the NARCOBIT-E anesthesia device (Natsume Seisakusho Co., Ltd. Tokyo, Japan). For immunohistochemistry, the pups were transcardially perfused with 4% paraformaldehyde (PFA) prepared in 1X PBS and brains were dissected at P0, P3, P5, P10, and P20 and post-fixed overnight in 4% PFA at 4 °C. Brains were washed three times for 10 min each with PBS, then dehydrated with 80% ethanol and kept until used. For Western blot analysis, the brains of C57BL/6 mice at P10 and P20 were freshly dissected into the cerebral cortex, brainstem, basal ganglia, and cerebellum, then directly processed for Western blotting. The cerebellums was not collected from P0 and P5 mouse brains due to their small size.

### 2.4. Western Blot

The freshly dissected tissues were extracted in RIPA buffer (50 mM Tris-HCl Buffer pH 7.6, 150 mM NaCl, 1% Nonidet P40 Substitute, 0.5% Sodium Deoxycholate, 0.1% sodium dodecyl sulfate (SDS)). Total protein lysates were prepared by homogenization in solution using a plastic pestle and cleared by centrifugation at 16,000 g at 4 °C for 10 min. Protein concentration was determined using nanodrop (Thermo Fisher; ND-ONE-W). An equal amount of total protein was loaded in 8% and 12% SDS/PAGE gel and transferred to PVDF membranes (PALL BSP0161; FUJIFILM, Tokyo, Japan). The membranes were immersed in blocking buffer (4% skim milk, 1× Tris-buffered saline, 0.1% Tween 20 (TBST)) for 10 min, then incubated with primary antibodies diluted in the blocking buffer overnight at 4 °C. After washing with TBST, the membranes were incubated at room temperature for 1 h with donkey anti-rabbit polyclonal antibody (1:5000) diluted in blocking buffer. Membranes were washed three times with TBST and scanned using a chemiluminescence imaging system (FUSION SOLO S; Vilber Inc., Collégien, France). Primary antibodies used for Western blot are the rabbit anti-Sbno1generated in this study (1:1000) and rabbit anti-β-actin (4967S, 1:1000, Cell Signaling Technology, Danvers, MA, USA). Western blot signals of Sbno1 expression were measured using ImageJ 1.53 and samples were consistently normalized to β-actin across all stages and brain regions. This normalization was applied uniformly to all samples to ensure accurate comparisons between experimental conditions and time points. A figure showing the normalization process using β-actin as an internal control is provided as Supplementary Data.

### 2.5. Brain Section Preparation and Immunostaining

The three mouse brains were sectioned and stained using anti-Sbno1 antibody. Because we obtained reproducible results, two tissue sections were analyzed in each experimental condition quantitatively (Supplementary Table). The fixed brains were embedded in paraffin utilizing an automatic embedding system (Tissue-Tek VIP5; Sakura Finetek Japan Co., Ltd., Tama, Japan) and were sectioned using a microtome (Leica SM 2010 R, Leica Microsystems, Wetzlar, Germany) at a thickness of 7 µm. Paraffin sections were deparaffinized with xylene and hydrated with a dilution series of ethanol. Sections were autoclaved with Tris-EDTA (pH 9.0) for antigen retrieval. Subsequently, they were rinsed with PBS three times for 10 min each and incubated in blocking buffer (1% BSA, 3% TritonX-100, 1% PSA in PBS) for 1 h. The antibodies were diluted with blocking buffer. The sections were incubated with the primary antibody overnight at 4 °C. After washing with PBS three times for 10 min each, the sections were incubated for 1 h at room temperature with fluorescent secondary antibody (see below). After washing with PBS, the sections were mounted using a DAKO fluorescence mounting medium (S3023, Agilent, Santa Clara, USA) and coverslipped.

The primary antibodies used in this study are anti-Sbno1 antibody (1:1000) and rat anti-Ctip2 (abcam ab18465; 1:1000) or mouse anti-CPNase (Merck MAB326; 1:200) or goat anti-IBA (abcam ab5076; 1:1000) or mouse anti-GFAP (abcam ab4674; 1:1000) or mouse anti-CD133 (abcam ab264545; 1:500) antibodies. After washing, sections were incubated with DAPI (Thermo Fisher Scientific, D1306), anti-rabbit IgG Alexa Fluor 488 (1:1000) and anti-rat IgG secondary Alexa Fluor antibody (Thermo Fisher Scientific A-21434) or anti-mouse IgG secondary antibody Alexa Fluor 555 (Thermo Fisher Scientific, A-31570; 1:1000) or anti-mouse IgG secondary antibody Alexa Fluor 555 (ThermoFisher Scientific A-21432; 1:1000).

### 2.6. Microscopy and Image Analysis

All fluorescent images were taken with a Leica DMi8 confocal microscope. Bright-field images were captured with the All-In-One microscope (BZ-X710; Keyence, Osaka, Japan). Images were acquired from anatomically matched coronal sections along the rostral–caudal axis. Brain anatomy was identified using the Allen Brain Atlas (https://atlas.brain-map.org/ accessed on February 2024) and the mouse brain in stereotaxic coordinates [15]. The intensity of Sbno1 immunofluorescent signal was measured in various brain regions and their neuronal populations using ImageJ. Regions of interest (ROIs) were selected by identifying 8 cells that expressed Sbno1 within each brain region of interest. These cells were chosen based on their distinct boundaries relative to the background. Fluorescence intensity was quantified within these ROIs, and care was taken to select cells representative of the overall region. The same criteria were applied consistently across all samples to ensure reproducibility and reduce selection bias.

## 3. Results

### 3.1. Quantitative Examination of Sbno1 Protein Level in the Different Brain Regions

**Figure 1:**
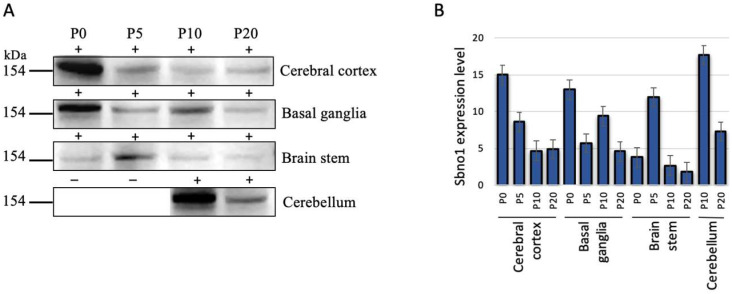
The expression levels of Sbno1 vary quantitatively across four major brain regions during postnatal stages (P0, P5, P10, and P20). (**A**) Western blot shows developmentally regulated Sbno1 expression in the cerebral cortex, basal ganglia, brainstem, and cerebellum. The cerebellum could not be dissected at P0 and P5, so Sbno1 protein levels were examined at P10 and P20. Equal amounts of total protein were loaded for each sample. The major bands at predicted molecular size are shown. (**B**) The graph shows a quantitative comparison of Western blot signal intensities expressed as arbitrary units. (**A**,**B**) In the cerebral cortex, the Sbno1 protein level was highest at P0 and progressively declined by P20. In the basal ganglia, the Sbno1 protein level was high at P0, decreased at P5, increased again at P10, and then declined by P20. In the brainstem, Sbno1 protein levels were weak at P0, P10, and P20, with a transient increase observed at P5. For the cerebellum, analyzed at P10 and P20 due to its later development, the Sbno1 protein level was high at P10 and P20. + label indicates the presence of the sample and – label suggests the absence of the sample.

The brains were dissected into the cerebral cortices, basal ganglion, and brainstem to quantitatively examine Sbno1 protein levels. Since the cerebellum becomes macro-anatomically apparent at P10, it was only included in dissection at both P10 and P20. Overall, our antibody detected one major band at predicted size and two minor bands at positions higher than 100 and 75 kDa in Western blot in all brain regions at all postnatal stages (Figure S2). An increase and decrease in the intensity of these three bands were consistently declined along postnatal development (Figure S2), suggesting that all three bands are isoforms of Sbno1. Sbno1 expression showed dynamic changes across these brain regions during postnatal development (Figure 1). In the cerebral cortex, the Sbno1 protein level was high at P0 followed by a progressive decline by P10. No significant differences in Sbno1 protein levels were observed between P10 and P20. Together with our previous findings, which showed an increase in Sbno1 protein expression during embryogenesis [14], these results suggest that the Sbno1 protein level in the cerebral cortex peaks perinatally, decreases after birth, and stabilizes at a constant level in later stages. In the basal ganglia, Sbno1 protein levels were high at P0, dropped at P5, increased again at P10, then decreased at P20 (Figure 1). In the brainstem, Sbno1 protein levels were weak at P0, P10, and P20, with a transient increase observed at P5 (Figure 1). For the cerebellum, which was examined at P10 and P20 when this region is sufficiently developed for dissection, the Sbno1 protein level was high at P10 and declined at P20 (Figure 1). Probably, a transient increase in Sbno1 expression in the brain stem at P5 attributed to Sbno1 expression in the presumptive cerebellar region. This quantitative analysis by Western blot revealed temporal changes in Sbno1 protein levels in four brain regions, suggesting that Sbno1 protein levels are developmentally regulated and vary between different brain regions.

### 3.2. Sbno1 Is Specifically Observed in Neurons in the Brain

**Figure 2:**
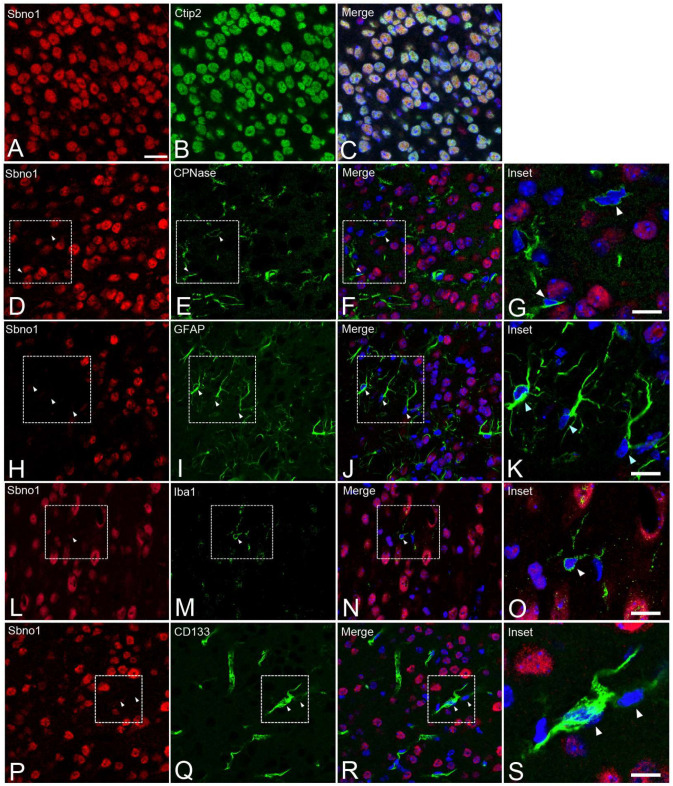
Simultaneous detection of Sbno1 (red) and various cell-type markers (green) in the cerebral cortex. All sections were counterstained with DAPI (blue). (**A**–**C**) Simultaneous detection of Sbno1 (red) and the cortical excitatory neuron marker Ctip2 (green). (**D**–**G**) Simultaneous detection of Sbno1 (red) and the oligodendrocyte marker CNPase (green). (**H**–**K**) Simultaneous detection of Sbno1 (red) and the astrocyte marker GFAP (green). (**L**–**O**) Simultaneous detection of Sbno1 (red) and the microglia marker Iba-1 (green). (**P**–**S**) Simultaneous detection of Sbno1 (red) and the vascular endothelial cell marker CD133 (green). (**G**,**K**,**O**,**S**) are enlarged images indicated by rectangles in (**F**,**J**,**N**) and (**R**), respectively. These results demonstrated that Sbno1 expression is restricted to neurons, with no expression observed in non-neuronal cells. Scale bar in (**A**) indicates 20 µm. Scale bars in (**G**,**K**,**O**,**S**) indicate 10 µm.

Our previous study identified the neuron-specific localization of Sbno1 during brain development [14]; however, preliminary experiments using our newly produced Sbno1 antibody exhibited high sensitivity, possibly raising the potential for overlooked signals in non-neuronal due to the lower sensitivity of the antibody that we used in the previous study [14]. To test this hypothesis, the cellular localization of Sbno1 was assessed in P10 cerebral cortex sections using Sbno1 double immunofluorescence with antibodies directed against neuronal, astrocytic, oligodendrocytic, endothelial, and microglial-specific markers. We confirmed Sbno1 expression in neurons by observing the colocalization of signals for Sbno1 and Ctip2, a marker for cortical excitatory neurons (Figure 2A–C). CNPase (Figure 2D–G), GFAP (Figure 2H–K), and Iba-1 (Figure 2L–O) were used as markers for astrocytes, oligodendrocytes, and microglia, respectively. We observed that all cells positive for these glial cell markers were negative for Sbno1. Vascular endothelial cells were identified by CD133 expression [16], and these cells also lacked Sbno1 expression (Figure 2P–S). Consistent with our previous observation [14], these observations indicated that Sbno1 is specifically expressed in neurons while non-neuronal cells do not express it.

### 3.3. Sbno1 Expression in the Cerebral Cortex

**Figure 3:**
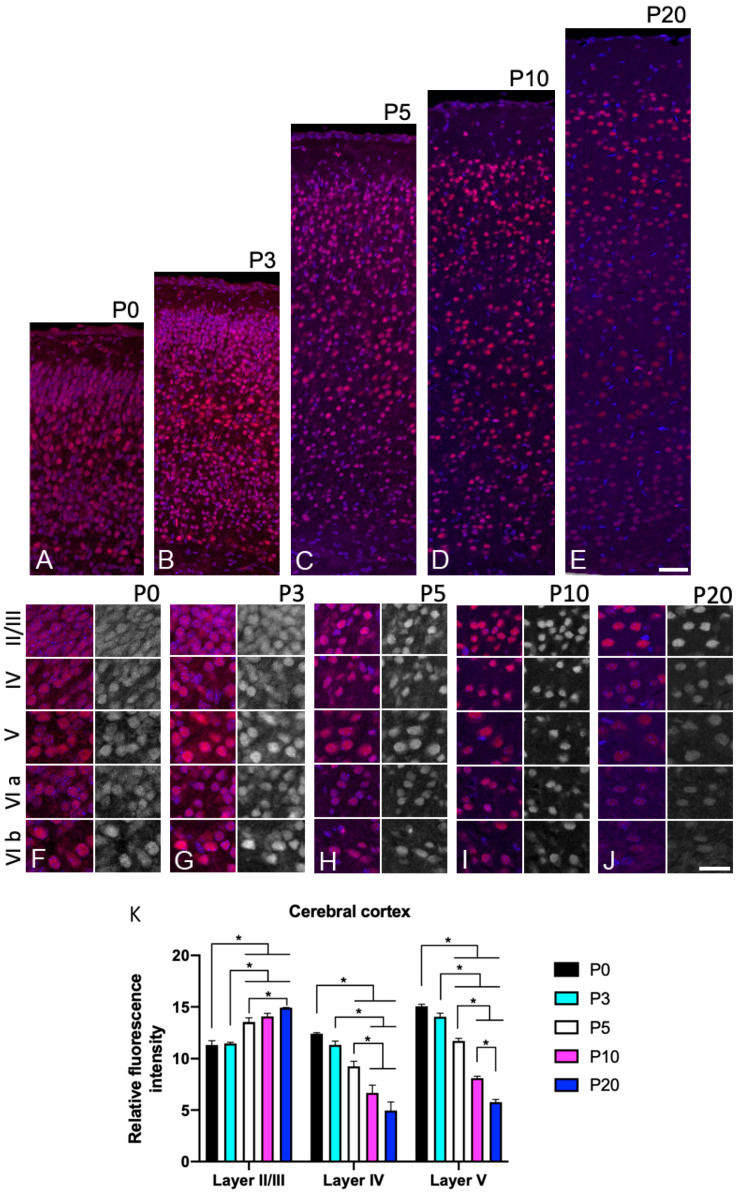
The changes in the expression level of Sbno1 expression during postnatal development are different in each cortical layer. Expression of Sbno1 (red) was detected in brain sections at P0, P3, P5, P10, and P20, as indicated. The sections were counterstained by DAPI (blue). (**A**–**E**) Lower magnification images of the cerebral cortices vertically from the ventricular side (**bottom**) to the pial surface (**top**). (**F**–**J**) Higher magnification images of cortical layers (II/III, IV, V, VIa, VIb). The left columns of the panels show merged images, and the right columns show black–white images of Sbno1 expression. Observations were made in the primary somatosensory area. Quantitative data (**K**) is shown. (**A**,**B**,**F**,**G**,**K**) At P0 and P3, Sbno1 immunoreactivity was strong in all neurons, with slightly higher levels observed in layer V and layer VIb neurons. (**C**,**H**) At P5, Sbno1 immunoreactivity in layer II/III neurons began to increase, a trend that continued at P10 (**D**,**I**,**K**). By P20, overall Sbno1 immunoreactivity in the cortex decreased, though it remained prominent in layer II/III (**E**,**J**,**K**). Scale bars in (**E**,**J**) indicate 50 µm and 20 µm, respectively. Statistical analysis of fluorescence intensity was performed by one-way ANOVA followed by Tukey’s multiple comparisons test (* *p* < 0.05).

The neocortex is a six-layered structure distinguishable by its cytoarchitecture, which can be visualized by DAPI staining. We investigated the expression pattern of the Sbno1 protein in the postnatal cortex from P0 to P20. Consistent with our Western blot results, immunohistochemical analysis for Sbno1 antiserum showed a decrease in Sbno1 immunoreactivity as cortical development progresses (Figure 1). Because anti-Sbno1 immunoreactivity showed a laminar pattern, we double stained the sections using anti-Sbno1 and anti-Ctip2 antibodies (Figure S3). Ctip2 is expressed in small neurons in layer VI and large neurons in layer V [17]. The cell-dense upper layer was defined as layer II/III. The cell-sparse layer between layers II/III and V was defined as layer IV. Sbno1 immunoreactivity was strong in all neurons but slightly higher in layer V and layer VIb neurons at P0 and P3 (Figure 3A,B,F,G,K). Sbno1 immunoreactivity in layer II/III started to increase at P5 (Figure 3C,H) and this trend continued at P10 (Figure 3D,I,K). At P20, overall Sbno1 immunoreactivity in the cortex became weaker, although it remained prominent in layer II/III (Figure 3E,J,K). Throughout all developmental stages examined, we observed heterogeneity in Sbno1 immunoreactivity among neurons. This heterogeneity did not appear to correspond to particular neuronal subtypes, such as inhibitory or excitatory neurons, as Sbno1 immunoreactivity was also observed within the neurons devoid of Ctip2 immunoreactivity within the layer V of the cerebral cortex (Figure 2A,C).

When examining Sbno1 immunoreactivity across serial sections from anterior to posterior regions of the cerebral cortex, we found that Sbno1 immunoreactivity was notably more prominent in the piriform cortex compared to other cortical regions. Cells sporadically distributed in layer III of the piriform cortex exhibited robust Sbno1 expression, and their density transiently increased at P3 (Figure S4). Sbno1 immunoreactivity in layer II also increased transiently at P3 and was maintained at a moderate level in later stages. Layer I contained few strongly Sbno1-immunoreactive cells until P10 and by P20, such cells were scarcely observed (Figure S4). The distinct and complex Sbno1 immunoreactive pattern during the hippocampal development (Table 1) will be reported elsewhere, as it exhibited unique spatial and temporal characteristics (Zolzaya and Katsuyama, in preparation). In summary, the immunoreactivity for Sbno1 changes dynamically during postnatal brain development, with distinct patterns across cortical layers. Furthermore, neurons exhibit heterogeneity in Sbno1 immunoreactivity within the layer.

### 3.4. Sbno1 Expression in the Olfactory Bulb

**Figure 4:**
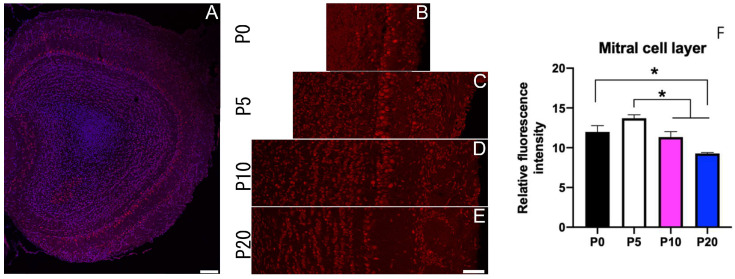
Expression of Sbno1 in the olfactory bulb in the postnatal brains. (**A**) A whole section of the olfactory bulb indicating Sbno1 expression (red) and nuclei (blue). (**B**–**E**) Expression of Sbno1 in the laminar structure of the olfactory bulb through indicated postnatal developmental stages. Quantitative data (**F**) is shown. Cells in the glomerular and external plexiform layers exhibited weak and consistent Sbno1 immunoreactivity throughout development (**A**–**E**). Prominent Sbno1 immunoreactivity was observed in the mitral cell layer, with the number of immunoreactive cells increasing at P5 (**C**,**F**), followed by a gradual decrease at later stages (**D**–**F**). Scale bars in (**A**,**E**) indicate 100 µm and 50 µm, respectively. Statistical analysis of fluorescence intensity was performed by one-way ANOVA followed by Tukey’s multiple comparisons test (* *p* < 0.05).

The olfactory bulb has a layered cytoarchitecture with a distinct pattern of Sbno1 immunoreactivity. Cells in the glomerular and external plexiform layers were weakly immunoreactive for Sbno1 and this remained consistent over time (Figure 4). Prominent Sbno1 immunoreactivity was observed in the mitral cell layer, where the number of Sbno1 immunoreactive cells increased at P5 (Figure 4C,F), then gradually decreased thereafter (Figure 4D–F). In contrast, only a few granular cells displayed strong Sbno1 immunoreactivity at P0, but the proportion of granular cells immunoreactive to Sbno1 increased as development progressed. These observations suggested that the temporal changes in Sbno1 immunoreactivity were distinct in the mitral cell and the granular cell layers during the postnatal development of the olfactory bulb.

### 3.5. Sbno1 Expression in the Cerebellum

**Figure 5:**
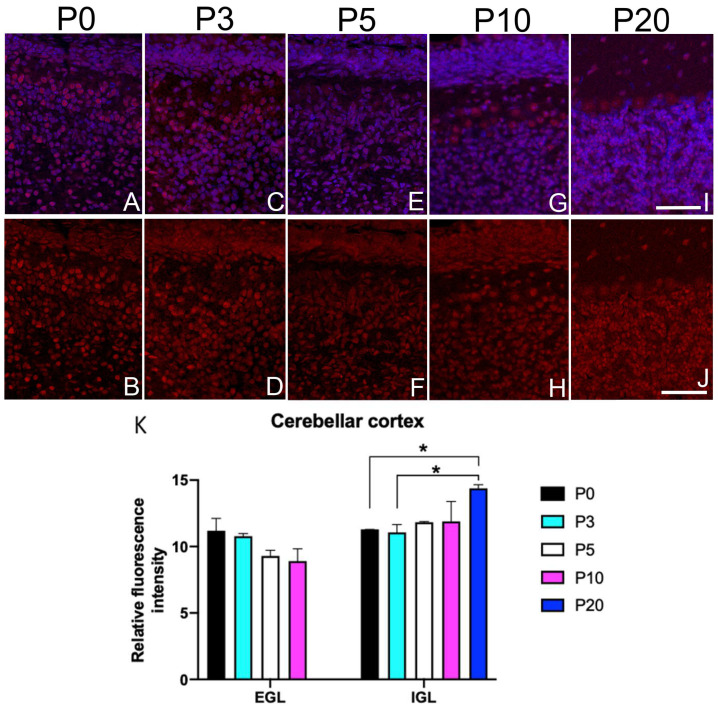
Sbno1 immunoreactivity in the postnatal cerebellar cortex. Sbno1 immunoreactivity is heterogeneous, cells in the external granular layer show a weak Sbno1 immunoreactivity, while a significant fraction of cells in the internal granular layer displays a strong Sbno1 immunoreactivity. (**A**,**C**,**E**,**G**,**I**) Merged images of Sbno1 immunofluorescence (red) and DAPI staining (blue). (**B**,**D**,**F**,**H**,**J**) Images of Sbno1 immunofluorescence only (red). Quantitative data (**K**) are shown. (**A**–**H**,**K**) In the external granule layer (EGL), Sbno1 immunoreactivity was weak but homogenously observed from P0 to P10. In contrast, the internal granule layer (IGL) displayed heterogeneous Sbno1 immunoreactivity over time (**A**–**K**)**.** By P20, granule cells showed a similar intensity of Sbno1 immunoreactivity and tended to form clusters (**I**–**K**). At P20, Sbno1 was also observed in the molecular layer (**I**,**J**), while Purkinje cells exhibited weaker Sbno1 immunoreactivity compared to granule cells (**G**,**I**,**K**). Scale bars in (**I**,**J**) indicate 100 µm. Statistical analysis of fluorescence intensity was performed by one-way ANOVA followed by Tukey’s multiple comparisons test (* *p* < 0.05).

After birth, the cerebellar surface consists of the external granular layer (EGL), where cells proliferate and migrate tangentially, then radially to form the internal granular layer (IGL), where they differentiate to establish local networks. Concurrently, as granule neurons proliferate, Purkinje neurons arborize their dendrites in the cerebellar cortex molecular layer, forming synapses with the parallel fibers of the granule cells and some types of inhibitory neurons. In the EGL, Sbno1 immunoreactivity was weak but homogenously observed from P0 to P10 (Figure 5A–H,K). In contrast, cells in the IGL showed heterogeneous Sbno1 immunoreactive intensity over time (Figure 5). By P20, granule cells that have a similar intensity of immunolabeling for Sbno1 tend to form clusters (Figure 5I–K). Again at P20, the molecular layers which are composed of inhibitory neurons showed Sbno1 immunoreactivity (Figure 5I,J), while Sbno1 immunoreactivity in Purkinje cells was weaker than in the granule cells (Figure 5G,I,K).

### 3.6. Sbno1 Expression in the Brainstem

**Figure 6:**
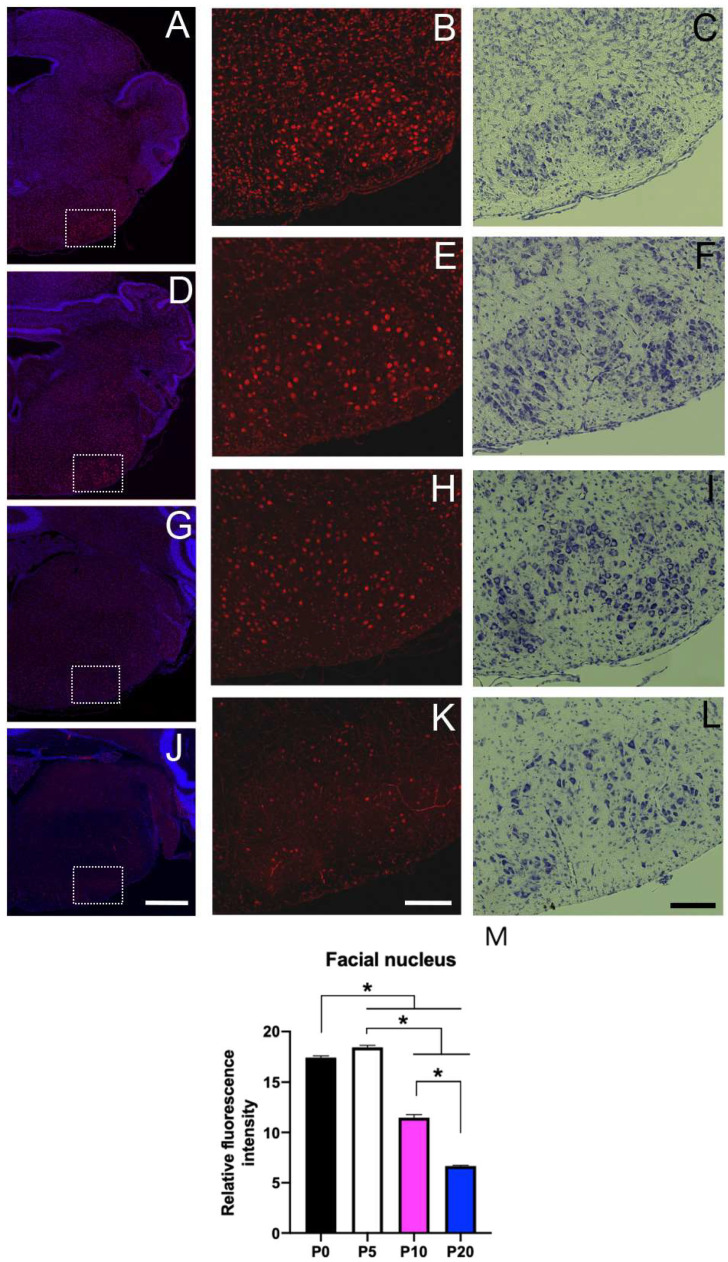
Prominent expression of Sbno1 in the oculomotor nucleus and red nucleus at P0 (**A**,**B**), P5 (**D**,**E**), P10 (**G**,**H**), and P20 (**J**,**K**). (**A**,**D**,**G**,**J**) Merged images of Sbno1 expression (red) and DAPI (blue) in the coronal section at the superior colliculus level at a low magnification. (**B**,**E**,**H**,**K**) Images of immunoreactivity indicating Sbno1 expression at a higher magnification of the region indicated by rectangles in (**C**,**F**,**I**) and (**L**), respectively. (**C**,**F**,**I**,**L**) are bright-field images showing Nissl staining of the adjacent sections of (**B**,**E**,**H**) and (**K**), respectively. Quantitative data (**M**) are shown. (**B**,**E**,**H**,**K**,**M**) Some neurons in the oculomotor nucleus exhibited strong Sbno1 immunoreactivity. (**B**,**E**,**H**,**K**) A similar pattern of strong Sbno1 immunoreactivity was observed in neurons of the magnocellular part of the red nucleus. A scale bar in (**J**) indicates 200 µm. Scale bars in (**K**,**L**) indicate 100 µm. Statistical analysis of fluorescence intensity was performed by one-way ANOVA followed by Tukey’s multiple comparisons test (* *p* < 0.05).

**Figure 7:**
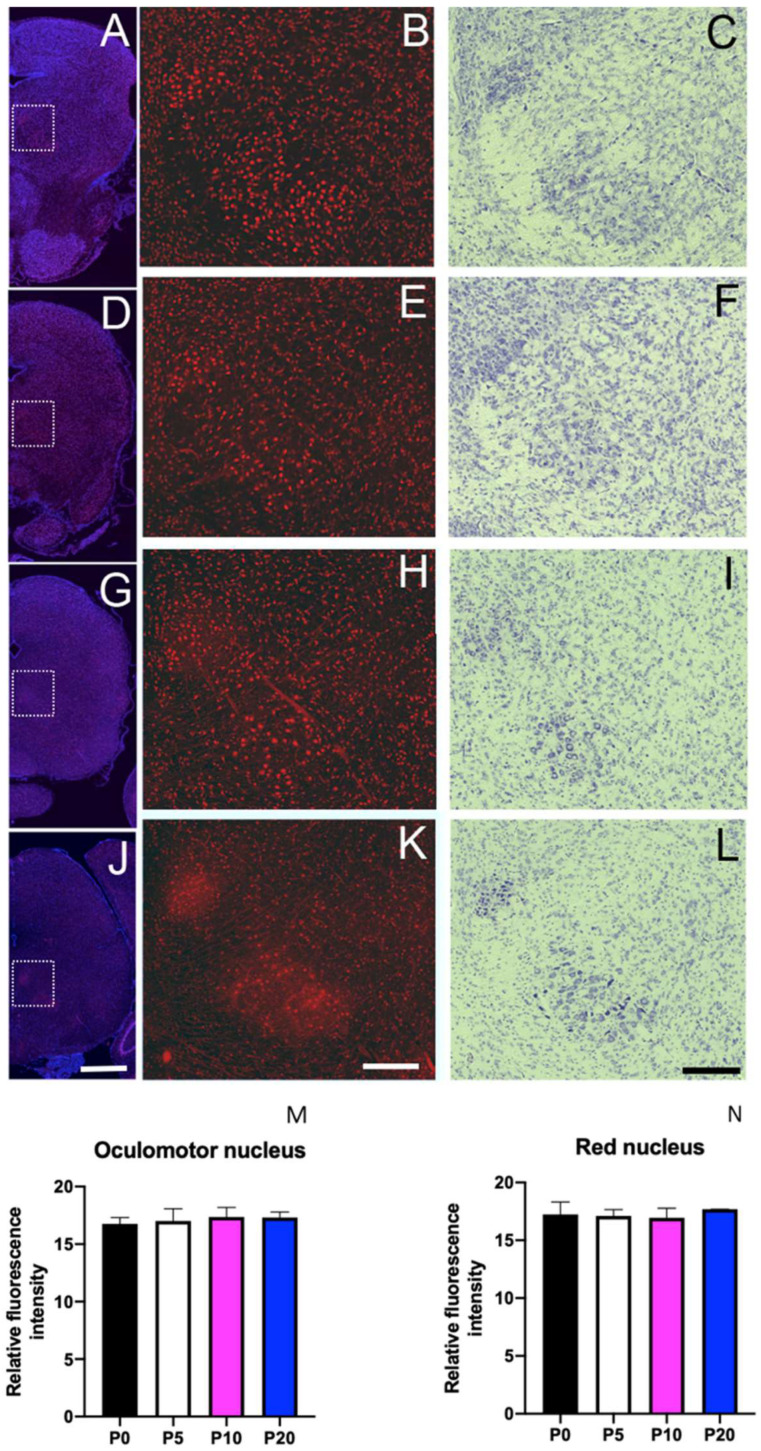
Prominent Sbno1 immunoreactivity in the facial nucleus at P0 (**A**,**B**), P5 (**D**,**E**), P10 (**G**,**H**), and P20 (**J**,**K**). (**A**,**D**,**G**,**J**) Merged images of Sbno1 immunofluorescence (red) and DAPI (blue) in the coronal section at the upper medulla level, displayed at a low magnification (**B**,**E**,**H**,**K**). Higher magnification images of SBNO1 immunoreactivity in the region outlined by rectangles in (**A**,**D**,**G**) and (**F**), respectively. (**C**,**F**,**I**,**L**) Bright-field images of Nissl staining in adjacent sections corresponding to the sections shown in (**B**,**E**,**H**) and (**K**), respectively. Quantitative data of intensity of immunoreactivity in the oculomotor nucleus (**M**) and the red nucleus (**N**) are shown. At P0, Sbno1 immunoreactivity was stronger in the facial nucleus compared to surrounding neurons (**B**,**M**). The intensity of Sbno1 immunoreactivity in the facial nucleus peaked at P5 (**E**,**M**) and gradually declined by P10 (**H**,**M**), with only a few cells retaining strong immunoreactivity at P20 (**K**,**M**). A scale bar in (**J**) indicates 200 µm. Scale bars in (**K**,**L**) indicate 100 µm.

We observed Sbno1 immunoreactivity expression throughout the brainstem, though neurons with strong Sbno1 immunoreactivity were sporadically observed. By comparing Nissl-stained adjacent sections, we identified the cells with sporadic strong Sbno1 immunoreactivity to have the characteristic of motor neurons morphology [18]. In the sections at the level of the superior colliculus, some (but not all) neurons in the oculomotor nucleus displayed strong Sbno1 immunoreactivity (Figure 6), and similar strong Sbno1 immunoreactivity was observed in some neurons of the magnocellular part of the red nucleus (Figure 6). These immunoreactive patterns of Sbno1 persisted throughout postnatal development, and similar strong immunoreactivity of Sbno1 was observed in the facial nucleus at the levels of the upper medulla (Figure 7). At P0, Sbno1 immunoreactivity was stronger in the facial nucleus compared to neurons in the surrounding regions (Figure 7B,M). The intensity of Sbno1 immunoreactivity in the facial nucleus peaked at P5 (Figure 7E,M) and gradually declined at P10 (Figure 7H,M), with only a few cells retaining strong Sbno1 immunoreactivity at P20 (Figure 7K,M). A similar temporal pattern of Sbno1 immunoreactivity was also observed in the trigeminal motor nucleus (Figure S5) and the hypoglossal nucleus (Figure S6). Moderate Sbno1 immunoreactivity, slightly stronger than that in the surrounding regions, was noted in the inferior olivary nucleus (Table 1). Strong immunoreactivity for Sbno1 was also sporadically detected in the reticular formation throughout the brainstem over time. We extensively examined Sbno1 localization in immunohistologically stained coronal sections throughout three brains at each postnatal stage. A quantitative comparison of Sbno1 immunofluorescence levels across brain regions and neuronal populations is summarized in Table 1.

**Table 1:** Summary of Sbno1 protein expression during postnatal period.

Region	Stage				
	P0	P3	P5	P10	P20
**Olfactory bulb**					
Granule cell layer	+	+	+	+	+
Internal plexiform layer	+	+	+	+	+
Mitral cell layer	++	++	+++	++	++
External plexiform layer	+	+	+	+	+
Glomerular layer	+	+	++	++	++
**Cerebral cortex**					
Layer I	±	±	±	±	±
Layer II/III	++	++	++	+++	+++
Layer IV	++	++	++	+	+
Layer V	+++	+++	++	++	+
Layer VIa	++	++	++	++	+
Layer VIb	+++	+++	+	+	+
**Hippocampus**					
Dentate gyrus					
Molecular layer	±	±	±	±	±
Granule cell layer	+	+	+	+	++
Hilus	++	++	++	++	+
CA3					
S. oriens	±	±	±	±	±
S. pyramidale	+++	+++	+++	+++	+++
S. radiatum	±	±	±	±	±
CA1					
S. oriens	±	±	±	±	±
S. pyramidale	++	++	++	++	++
S. radiatum	±	±	±	±	±
**Piriform cortex**					
Layer I	+	+	+	+	+
Layer II	++	++	++	++	++
Layer III	+++	+++	+++	+++	+++
**Basal ganglia**	++	+	+	+	+
**Thalamic nuclei**	+	+	+	+	+
**Superior colliculus**	+	+	+	+	+
**Red nucleus**	++++	++++	++++	++++	++++
**Inferior colliculus**	+	+	+	+	+
**Cerebellum**					
External granular layer	+	+	+	+	
Molecular layer					++
Purkinje cell layer	+	+	+	++	++
Internal granular layer	+	++	++	+++	+++
Deep cerebellar nucleus				++	++
**Pontine nucleus**	+	+	+	+	+
**Inferior olivery nucleus**	++	++	++	++	++
**Dorsal cochlear nucleus**	+	+	+	+	+
**Vestibular nucleus**	+	+	+	+	+
**Motor nuclei of brainstem**					
Oculomotor nucleus	++++	++++	++++	++++	++++
Facial nucleus	+++	++++	++++	+++	+
Trigeminal motor nucleus	++++	++++	++++	++++	++
Hypoglossal nucleus	+++	++++	++++	++++	++++

This table summarizes the Sbno1 protein expression levels across various brain regions and cortical layers during the postnatal stages (P0, P3, P5, P10, and P20). As far as we observed, all neurons express Sbno1. Number of + indicates relative intensity of Sbno1 immunoreactivity in each neuronal structure or cell type. ± indicates that cells expressing Sbno1 were significantly observed in the regions but density of the cells was low. Layer VIa and VIb refer to the sublayers of layer VI in the cerebral cortex. Data are derived from immunohistochemical analyses and represent consistent patterns observed across biological replicates.

## 4. Discussion

Sbno1 is a DExD/H box helicase belonging to superfamily 2, and most DExD/H box helicases are known to function as RNA helicases in the cytoplasm [19,20]. In the present study, we observed nuclear localization of Sbno1 in neurons, consistent with our previous findings that Sbno1 interacts with Rbpj, a DNA-binding transcription factor [13]. This suggests that DExD/H box helicases may function both in the cytosol and/or cell nucleus, recognizing both DNA and RNA, likely depending on the combination of functional motifs within the amino acid sequence of the protein. The Sbno1 immunoreactivity in the neuronal nucleus aligns with the fact that the Sbno1 protein contains two nuclear localization signals. However, Sbno1 immunoreactivity was observed not only overlapping with DAPI staining but also weakly detected in other cellular structures, such as neuronal fiber (Figure 6).

Genetic studies in *Drosophila* identified strawberry notch protein as a component of the Notch signaling pathway. Despite the involvement of the Notch signaling pathway in regulating myelination during postnatal brain development [21,22,23], it is unlikely that Sbno1 is involved in myelination, as it is specifically expressed in neurons and not in oligodendrocytes (Figure 2). The knockout (KO) of *Sbno1* in cortical neurons reduced both axonal growth and dendritic arborization [14]. Notch signaling has been shown to positively regulate dendritic branching while negatively regulating the dendritic growth of the primary cultured cortical neurons [24]. In neurons of the *p53* KO mouse, Notch signaling is hyperactivated, resulting in reduced neurite growth, further suggesting its role in neuronal plasticity [25]. This is consistent with earlier observations that the upregulation of Notch activity either inhibited extension or caused the retraction of neurites in the primary cultures of the cortical neurons [26]. While studies in *Sbno1* KO mice align with Notch function in dendritic branching, these studies diverge in conclusions regarding dendritic and axonal elongation.

On the other hand, histological studies in *Sbno1* KO [14] and *Rbpj* KO [27] model mice highlight distinct cytoarchitectural abnormalities. *Rbpj* KO mice exhibit a highly disorganized laminar structure in the cerebral cortex [27], whereas cortical lamination remains intact in *Sbno1* KO mice [14]. Thus, the radial migration of differentiating neurons in the embryonic cortex involves a Sbno1-independent Notch signaling pathway. Additionally, *Rbpj* KO mice survive until adulthood [27], whereas *Sbno1* KO mice die around three weeks after birth (unpublished). This suggests that Sbno1 may have unrevealed roles beyond its involvement in the Notch signaling pathway in postnatal neuronal development.

We also observed prominent Sbno1 expression in the olfactory bulb and the piriform cortex, which are regions involved in the odor integrate encoding/processing system. The piriform cortex receives primary afferent monosynaptic input from the olfactory bulbs’ projection neurons, including mitral and tufted cells. Given the direct connection between olfactory bulb and piriform cortex, Sbno1 may contribute to the development of the olfactory neuronal system pathway and sensory processing. This conclusion, along with multiple studies linking *SBNO1* to schizophrenia [3,5,9], may explain why schizophrenic patients have olfactory dysfunction [28].

In the brainstem, we found only a few neurons in several neuronal nuclei with prominent Sbno1 immunoreactivity. Although these neurons are generally large, an examination of Nissl-stained adjacent sections revealed that not all large neurons exhibit strong Sbno1 immunoreactivity, indicating that neuronal size is not a sufficient phenotype to identify Sbno1-expressing neurons. Indeed, a few large neurons in the sensory nuclei also showed a strong Sbno1 immunoreactivity. The oculomotor, facial, trigeminal motor, hypoglossal nuclei, and the ventral horn of the spinal cord are motor-related regions, and all contain large neurons that exhibit strong Sbno1 immunoreactivity. A comparison of Nissl-stained adjacent sections suggested that these are motor neurons. Interestingly, the layer V neurons in the cerebral cortex, which contribute to the pyramidal tract projecting to the motor neurons in the spinal cord, also displayed a strong Sbno1 immunoreactivity. Neurons with strong Sbno1 immunoreactivity were further observed in the magnocellular part of the red nucleus (Figure 6), and sporadically in the reticular formation. Since all these neuronal populations contain neurons projecting to the spinal motor neurons, Sbno1 may play a role in the differentiation and/or function of somatic motor pathways.

Our observations indicate that Sbno1 is expressed in all postnatal brain neurons, though the expression level varies depending on the neuronal population. Given that we previously showed that Sbno1 is involved in neurite growth [14], the expression level of Sbno1 may relate to neuronal morphogenesis, such as dendritic arborization, axonal growth, and synaptogenesis. The heterogeneous expression of Sbno1 suggests that Sbno1 expression could be regulated by neuronal activity, alike to immediate early genes, such as *c-Fos* [29]. Recently, a study found that the immediate early gene NPAS4 is involved in repairing DNA damage from neuronal activity [30]. As a helicase, Sbno1 could play a role in maintaining genomic stability, similar to other helicases [31,32]. Neurons, with their high metabolic demand and oxygen consumption leading to high oxygen species production, are prone to DNA damage and subsequent dynamic changes in gene expression [33]. The pan-neuronal expression of Sbno1 may thus be involved in genome stability. Since our results of in situ hybridization did not show heterogeneous staining in the brain section [34], Sbno1 expression is likely regulated post-transcriptionally.

In summary, our observations suggest that Sbno1 expression in neurons is developmentally regulated after birth. The histological observations of each brain region at different developmental stages are consistent with the quantitative comparison of Sbno1 expression levels by Western blot. Although Sbno1 is expressed in all neurons, the temporal and spatial pattern of the changes in intensity of Sbno1 immunoreactivity varies in different regions, including the olfactory bulb, cerebral cortex, cerebellum, motor nuclei in the brainstem, and unidentified sporadically distributed neurons. Therefore, Sbno1 likely has functions that are common to all neurons as well as specific roles in certain cell types and/or cellular activity, such as motor function of the brain. We previously showed that *Sbno1* knockout mice exhibited paralysis [14], which is consistent to the function of Sbno1 involved in motor function.

## Data Availability

Data are included in this MS.

## Supplementary Materials

The following supporting information can be downloaded at: https://www.mdpi.com/article/10.3390/jdb13010003/s1, Figure S1: Preparation of Sbno1 partial fragment. Validation of antibody specificity by Western blot; Figure S2: An example of full image of Western blot detecting Sbno1 Expression of beta-actin was examined as a internal control.; Figure S3: Simultaneous detection of Sbno1 and Ctip2 in the primary somatosensory cortex during the postnatal stages; Figure S4: Expression of Sbno1 in the piriform cortex in the postnatal brains; Figure S5: Prominent Sbno1 immunoreactivity in the trigeminal motor nucleus at P0, P5, P10, and P20; Figure S6: Prominent expression of Sbno1 in the hypoglossal nucleus at P0, P5, P10, and P20. Table S1: Provision of Quantification Data.
