# Supplementary material for: Neuronal Populations Involved in Motor Function Show Prominent Expression of Sbno1 During Postnatal Brain Development
Source: J Dev Biol. 2025 Jan 21;13(1):3. doi: 10.3390/jdb13010003 (PMC11843823; doi:10.3390/jdb13010003)
Supplement: Supplementary file 1 [file jdb-13-00003-s001.zip › jdb-3297620-supplementary/Supplementary materials last version/Supplementary figures with legends.pdf]

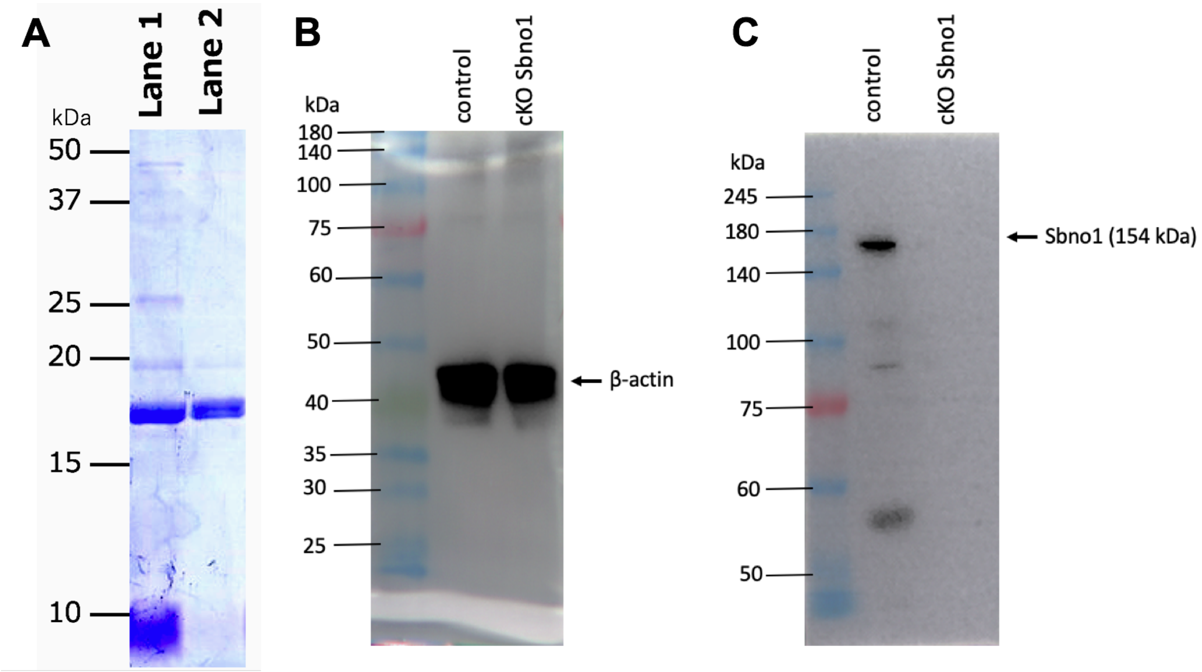

Figure S1.

Figure S1. (A) Lane1 loaded recombinant His-tagged partial fragment of SBNO1 protein detected by Coomassie brilliant blue staining. Lane 2 loaded final product of the fragment of SBNO1 protein from which His-tag was removed. (B) Expression of beta-actin in control and Sbno1 knockout cerebral cortices as a loading control. (C) Expression of Sbno1 in control and Sbno1 knockout cerebral cortices was detected using our newly constructed anti-Sbno1 antibody, showing specificity of the antibody. The major band was prominently detected at expected size. Additionally, three minor bands were observed in the control lane, but not in the lane of *Sbno1* knockout sample

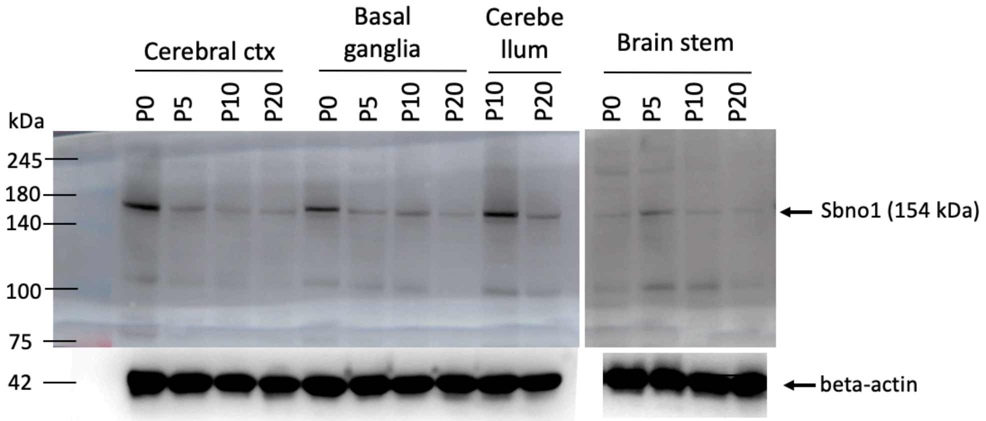

Figure S2. An example of full image of western blot detecting Sbno1 (upper panels) and beta-actin (lower panels). Only the major bands at the expected size (154kDa) were shown in Figure 1.

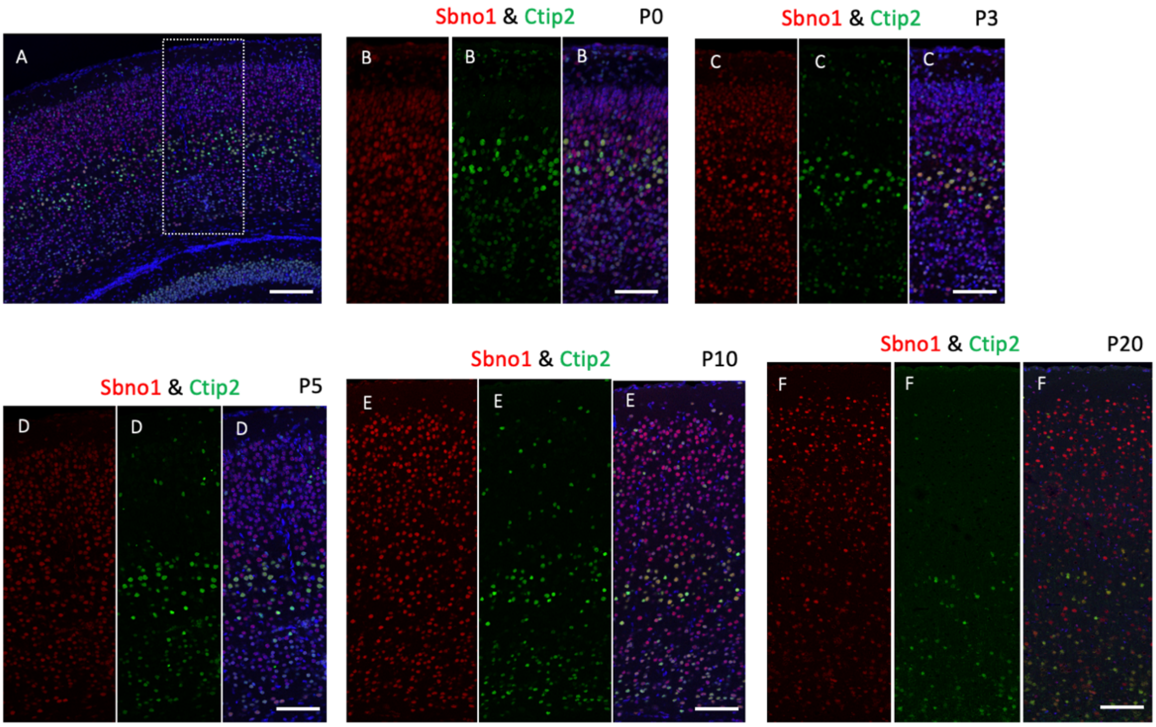

Figure S3. Simultaneous detection of Sbno1 and Ctip2 in the primary somatosensory cortex at P0 (B), P3 (C), P5 (D), P10 (E), and P20 (F). (A) Merged image of Sbno1 immunofluorescence (red), Ctip2 (green) and DAPI (blue) in the coronal section at a low magnification at P5. A scale bar in A indicates 100μm. Scale bars in B, C, D, E indicate 20μm. Ctip2 is strongly expressed in layer V, and weakly in layer VI. Cell-dense layer in the upper part of the cortex is layer II/III. We defined layer IV between layer II/III and layer V.

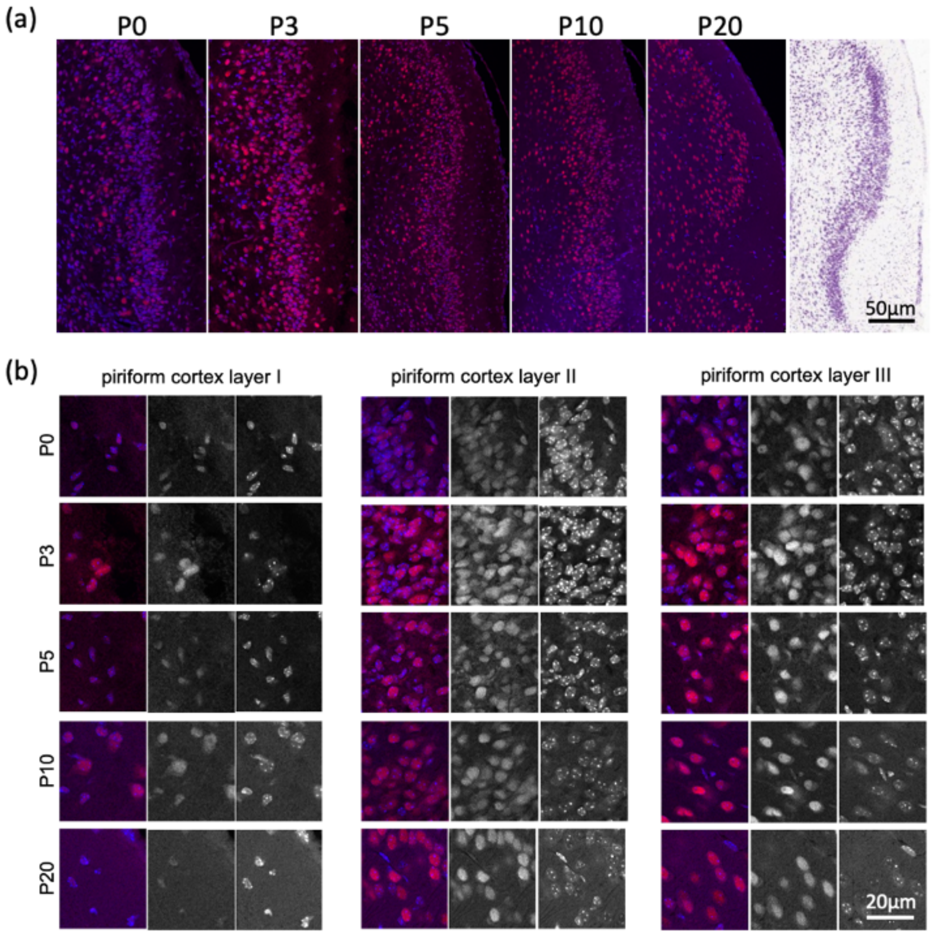

Figure S4. Expression of Sbno1 in the piriform cortex in the postnatal brains.
(a) The wide views of piriform cortex detecting Sbno1 immunohistochemically. Far right is a Nissl staining image of P5 piriform cortex. b) Sbno1 exhibits low expression levels in layer I of the piriform cortex during postnatal development. In contrast, its expression is notably elevated in layer II during developmental stages. The highest levels of Sbno1 expression are consistently observed in layer III of the piriform cortex across all developmental stages. Scale bars indicate 50μm (a) and 20μm (b).

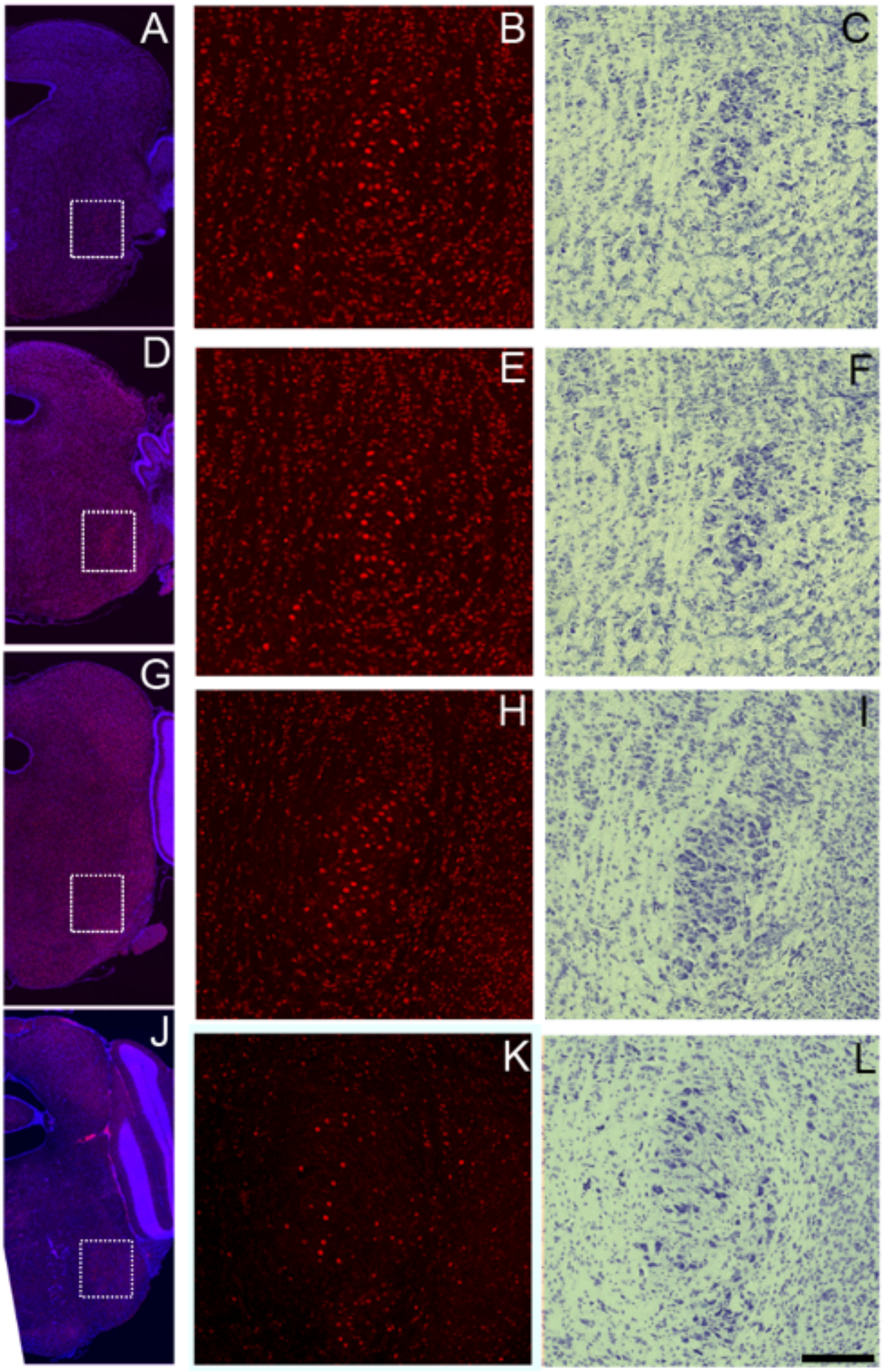

Figure S5. Prominent Sbno1 immunoreactivity in the trigeminal motor nucleus at P0 (A, B), P5 (D,E), P10 (G, H), and P20 (J,K). (A, D, G, J) Merged images of Sbno1 immunofluorescence (red) and DAPI (blue) at a low magnification. (B, E, H, K) Higher magnification images of SBNO1 immunoreactivity in the region outlined by rectangles in A, D, G, and F, respectively. (C, F, I and L) Bright field images of Nissl staining in the adjacent sections corresponding to the sections shown in B, E, H, and K, respectively. (B, E, H) From P0 to P10, Sbno1 immunoreactivity was stronger in the trigeminal motor nucleus than that in neurons in the surrounding area. (K) Only a few cells retaining strong immunoreactivity at P20. A scale bar indicates 100 μm.

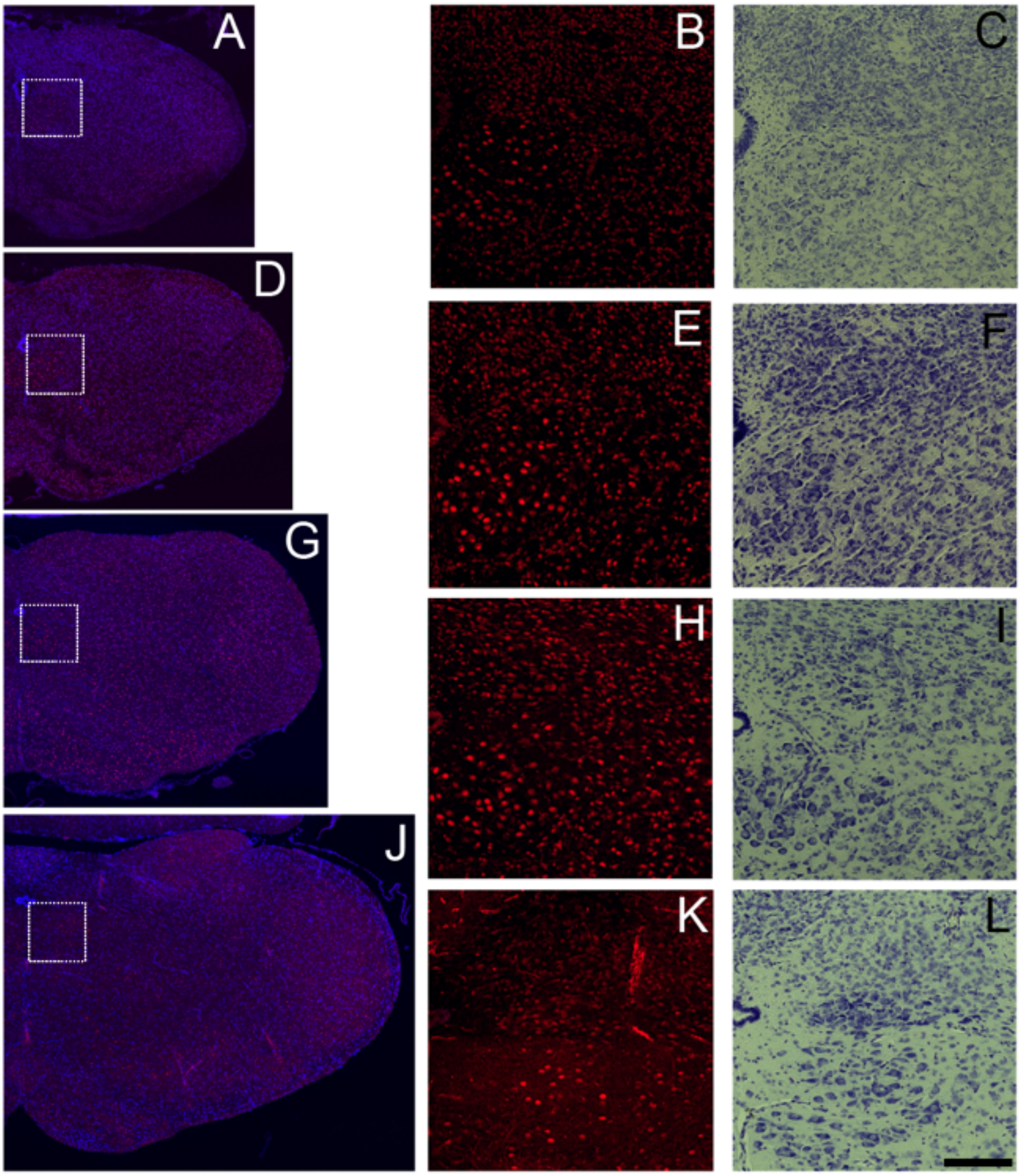

Figure S6. Prominent expression of Sbno1 in the hypoglossal nucleus at P0 (A, B), P5 (D,E), P10 (G, H), and P20 (J,K). (A, D, G, J) Merged images of Sbno1 expression (red) and DAPI (blue) at a low magnification. (B, E, H, K) Images of immunoreactivity indicating Sbno1 expression at a higher magnification of the region indicated by rectangles in C, F, I, and L, respectively. C, F, I, and L are bright field images showing Nissl staining of the adjacent sections of B, E, H, and K, respectively. The intensity of Sbno1 immunoreactivity in the hypoglossal nucleus peaked at P5 (E) and P10 (H), with only a few cells retaining strong immunoreactivity at P20 (K). A scale bar indicates 100 μm.

Supplementary table (Excel file)
Provision of Quantification Data: We have included all relevant quantification data, including raw intensity values for Western blot and immunofluorescence images, as supplementary Excel file. This file contains the raw data, statistical analyses, and p-values for all experiments.
